# Rise or fall? Reconciling contested directions in human gut hormone aging

**DOI:** 10.3389/fendo.2026.1952254

**Published:** 2026-09-07

**Authors:** Volodymyr Mavrych, Inna Shypilova, Olena Bolgova

**Affiliations:** 1 College of Medicine, Alfaisal University, Riyadh, Saudi Arabia; 2 School of Medicine, St. Matthew's University, George Town, Cayman Islands

**Keywords:** anorexia of aging, cholecystokinin, enteroendocrine cells, ghrelin resistance, glucose intolerance, incretin effect, nutrient sensing

## Abstract

Gut hormones govern appetite and postprandial glucose handling, and both functions deteriorate with age — yet the human literature cannot agree on the direction of the underlying hormonal changes. Incretin secretion is reported both to rise and to fall in older adults; ghrelin to decline, to be unchanged, and to be elevated; and the anorexia of aging is attributed variously to excess satiety signaling and to deficient basal hunger. This mini-review examines these three controversies, identifies the design features that give rise to them, and argues that they are largely reconcilable. Across all three, disputes about magnitude resolve when the outcome measured is instead the fidelity of coupling between nutrient ingestion and hormonal response: elevated GLP-1 coexists with an impaired incretin effect because it is compensation for β-cell failure, not preserved sensing; ghrelin concentrations may be normal while prandial rhythm is abolished; and older adults with low appetite show exaggerated anorexigenic responses despite unchanged subjective appetite. We further note that the human gut-hormone literature and the rapidly advancing rodent literature on age-related intestinal stem cell misdifferentiation have developed in near isolation, and that they currently disagree on whether the aged gut contains more or fewer enteroendocrine cells. Resolving these controversies requires studies that measure hormone concentrations and functional coupling in the same individuals, as well as longitudinal designs that extend beyond incretins.

## 1 Introduction

Enteroendocrine cells (EECs) are dispersed throughout the gut epithelium and collectively secrete more than twenty hormones in response to luminal and circulating cues (1, 2). Two clinical consequences of their age-related dysfunction are well recognized. The anorexia of aging (a physiological decline in appetite and energy intake in otherwise healthy older adults) predisposes to malnutrition and frailty. Separately, aging brings declining glucose tolerance and rising type 2 diabetes (T2D) incidence, in which the incretin axis is implicated.

**Figure 1:**
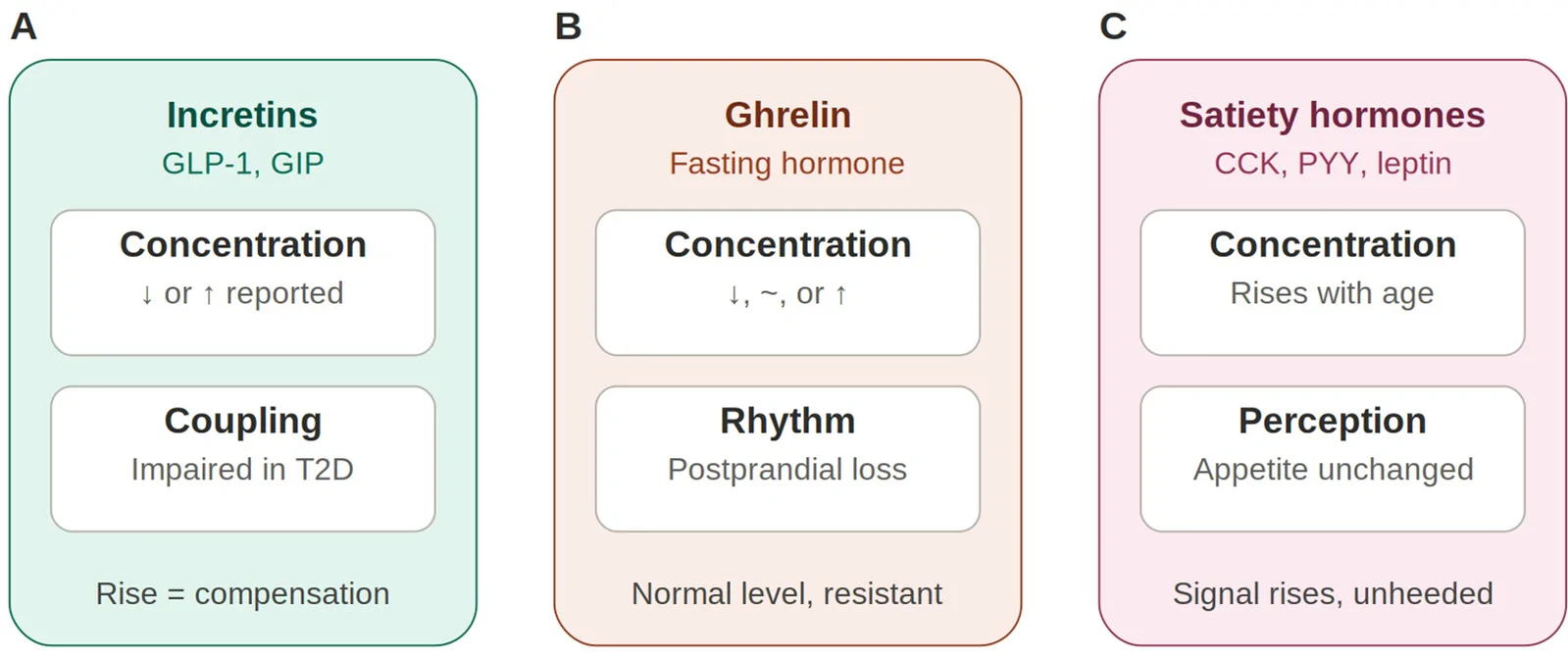
Concentration versus coupling across the three controversies. For each disputed hormone system, published reports disagree about whether concentration rises or falls with age (left), but converge once the outcome examined is the fidelity of coupling between the nutrient/feeding stimulus and the hormonal or physiological response (right). **(A)** Incretins: GLP-1 and GIP concentrations are reported as both reduced (7–10) and exaggerated (11, 12) with age; the incretin effect — the coupling between oral and intravenous glucose stimulation of insulin secretion is consistently impaired in T2D irrespective of concentration, and elevated GLP-1 is reconcilable as compensation for this impaired coupling rather than evidence of preserved sensing (13). **(B)** Ghrelin: mean fasting and postprandial concentrations are reported as decreased, unchanged, or increased (15, 17–19); postprandial rhythm (the fasted–fed excursion) is lost with age and frailty, and responsiveness to exogenous ghrelin is blunted, so a normal or elevated mean level can coexist with a fully decoupled or resistant system (19, 20). **(C)** Satiety hormones: CCK, PYY, and composite anorexigenic hormone scores rise with age (21–25), while subjective appetite does not track these changes proportionally, indicating a disturbance in signal transduction rather than in perception (21). Across all three panels, the same structural resolution applies: apparent disagreement about magnitude dissolves once concentration and coupling are treated as distinct, separately measurable properties.

What is far less settled is the direction of the hormonal changes underlying either. For nearly every gut hormone studied in humans, the literature contains credible reports pointing both ways. This review is organized around three such disputes. Our contention is that they are not merely unresolved but mis-specified: investigators have overwhelmingly measured hormone concentration, whereas the physiologically decisive variable is the fidelity with which secretion is coupled to nutritional state. A hormone can be abundant and uninformative. Recognizing this reconciles most of the apparent contradictions and identifies the experiments that would settle the remainder, as summarized schematically in **Figure 1**.

## 2 Materials and methods

Although Mini Reviews do not require a formal Materials and Methods section, we summarize our literature-search strategy for transparency. We searched PubMed, Scopus, and Web of Science for English-language articles published between January 2000 and June 2026, combining controlled-vocabulary and free-text terms. Reference lists of retrieved articles were screened for additional sources. Priority was given to peer-reviewed clinical studies, longitudinal and cross-sectional human cohort studies, systematic reviews/meta-analyses, and key rodent and Drosophila studies on intestinal stem cell differentiation; preprints, conference abstracts, and unpublished data were excluded.

Search string used: *(“aging” OR “ageing” OR “elderly” OR “older adults”) AND (“gut hormone” OR “enteroendocrine” OR “incretin” OR “GLP-1” OR “GIP” OR “ghrelin” OR “cholecystokinin” OR “peptide YY” OR “intestinal stem cell”) AND (“secretion” OR “concentration” OR “differentiation” OR “anorexia of aging” OR “glucose tolerance”)*.

## 3 The enteroendocrine sensor

EECs detect nutrients through electrogenic transporters such as SGLT1, ion channels, and nutrient-activated G protein-coupled receptors that converge on the exocytotic release machinery (3). Genetic support is direct: variants near *SLC5A1*, *GIPR*, *ABO*, and GLP2R are associated with circulating incretin concentrations, and several products have been confirmed in human EECs (4). Output is partitioned by nutritional state: fasting ghrelin is orexigenic, whereas postprandial GLP-1, GIP, PYY, and CCK promote satiety and glucose clearance. Enterochromaffin-cell serotonin adds a further layer: its release is nutrient- and fasting-modulated, and its peripheral actions notably include promoting hepatic glucose output rather than clearance (1). Because EEC subtypes are distributed unevenly along the proximal–distal axis, the region of nutrient exposure shapes the hormonal profile and metabolic response (5), and neuropod cells additionally relay luminal signals to vagal and primary sensory neurons via synapses (6). The system therefore performs an integrative, not merely secretory, function - which is why measures of integration behave differently from measures of amount.

We define functional coupling as the fidelity with which hormone secretion, or its downstream physiological action, tracks the nutrient or feeding stimulus over time: e.g., the amplification of insulin secretion by oral relative to intravenous glucose (the incretin effect; Section 4) or the fasted-to-fed excursion in circulating ghrelin (postprandial rhythm; Section 5) - as distinct from absolute hormone concentration, which reflects steady-state abundance independent of stimulus-response fidelity. This distinction underlies the reconciliation offered in each of the three controversies below.

## 4 Controversy 1: does incretin secretion rise or fall with aging?

The decline position rests on the strongest available design. Re-studying 41 healthy older subjects after ~5.9 years, fasting and glucose-stimulated GLP-1 both fell, fasting GIP fell, and gastric emptying slowed; the authors proposed this predisposes to glucose intolerance and T2D (7). This longitudinal design is the strongest available, though the follow-up cohort represented under half of the original 87 invited participants, introducing potential selection bias (7). Since almost all prior work was cross-sectional, this within-individual demonstration carries disproportionate evidential weight. Cellular data concur: elderly patients with T2D show reduced circulating GLP-1 and fewer colonic L-cells, and in aged high-fat/high-fructose rats, single-cell sequencing revealed downregulation of L-cell commitment factors, including Foxa1, alongside dysregulated GLP-1 processing, secretion, and nutrient-sensing genes (8). The human component of this study was cross-sectional (122 participants); the rat model provides mechanistic support that cannot be assumed to generalize directly to humans (8). In human tissue, GLP-1 and PYY release decline with age in the large, but not the small, intestine (9), and GLP-1 production is reduced in aged diabetic subjects (10). Both findings derive from cross-sectional human cohorts, surgical resection tissue in (9), and 50 non-obese subjects in (10), which cannot distinguish age-related decline from cohort or survivorship effects.

The elevation position is equally direct. Among 40 young and 53 older lean nondiabetic subjects, GLP-1 was elevated in both older groups irrespective of glucose tolerance (an exaggerated GLP-1 secretory response) yet insufficient to restore first-phase insulin release or overcome insulin resistance (11). Whey protein stimulated CCK and GIP more in older than younger adults (12). Both studies are cross-sectional and restricted to small, lean, nondiabetic samples [n = 93 in (11); 16 per age group in (12)], which (as discussed below) may itself explain part of the discrepancy with the decline literature rather than reflecting a true biological difference.

Three features reconcile these (**Table 1**). First, outcome measure: meta-analysis shows the incretin effect — amplification of insulin secretion by oral versus intravenous glucose (consistently impaired) in T2D versus normal glucose tolerance (weighted mean difference −27.3%), with age a significant modifier and fasting GIP an independent correlate (13), albeit with substantial between-study heterogeneity (I² = 86.6%), underscoring that this reconciling estimate is itself an average across disparate protocols (13). Elevated concentration and impaired coupling are therefore compatible; indeed, the former is expected as compensation for the latter. Second, cohort: elevation reports derive from lean, nondiabetic subjects (11), whereas decline reports are disproportionately from diabetic or unselected populations (8, 10). Third, stimulus: protein loads and glucose loads recruit different sensing pathways (3, 12), so age effects need not be uniform across macronutrients.

**Table 1:** Reported direction of change for each hormone system, and the variable that reconciles the disagreement.

Controversy	Reported as lower/unchanged	Reported as higher/exaggerated	Reconciling variable	Species/tissue
1. Incretin secretion (GLP-1, GIP)	Declines: fasting/stimulated GLP-1 and GIP fall longitudinally (7); fewer colonic L-cells and disturbed GLP-1 genes in aged T2D (8); GLP-1/PYY decline in aged human colon (9); reduced GLP-1 output in aged diabetics (10)	Elevated: exaggerated GLP-1 response in lean nondiabetic older adults (11); greater CCK/GIP response to whey protein in older adults (12)	Incretin effect (oral-vs-IV glucose coupling) is impaired in T2D independent of concentration; cohort (lean vs. diabetic) and stimulus (protein vs. glucose) explain divergent concentration reports (13)	Human (plasma/serum), except rat model in (8)
2. Ghrelin	Declines or unchanged: lower ghrelin in older normal-weight adults (15); no age difference in fasting/postprandial ghrelin (17); similar postprandial suppression across ages (18)	Elevated: raised ghrelin in klotho-deficient and SAMP8 aging mouse models (19); higher ghrelin in undernourished older women (18)	Loss of postprandial rhythm/suppression with age and frailty (20); ghrelin resistance despite elevated endogenous levels (19)	Human, except mouse models in (19)
3. Satiety hormones/appetite	Excess satiety: steeper CCK response to duodenal lipid in older men (22); exaggerated CCK-8-induced satiety, unaffected by supplementation (23, 24)	Deficient hunger: reduced basal hunger, not increased satiety, in undernourished older women (18)	Composite anorexigenic hormone score elevated in low-appetite older adults despite unchanged subjective appetite (21); meta-analysis shows elevated CCK/leptin/insulin/PYY with lower intake and hunger (25)	Human

What no study has done is measure concentration and functional coupling in the same aging individuals - the single experiment that would adjudicate this. L-cell vulnerability to lipotoxic stress under hypernutrition offers a plausible mechanism by which sustained compensation might eventually fail (14).

## 5 Controversy 2: ghrelin — falling levels, lost rhythm, or resistance?

Human reports split two ways, with rodent models suggesting a third. Ghrelin declines: plasma ghrelin in older normal-weight subjects is significantly lower than in the young and comparable to that in obesity, a pattern proposed to explain somatotroph dysregulation and elderly anorexia (15); this comparison involved small groups (older normal-weight n = 7) and was cross-sectional (15); concordant findings underpin ghrelin replacement work in aged mice (16). Ghrelin is unchanged: fasting and postprandial ghrelin did not differ between healthy elderly and young controls, where leptin and insulin (not ghrelin) tracked hunger and satiety (17); a second study found ghrelin fell similarly after preload across age groups, concluding that ghrelin changes are unlikely to be responsible for the anorexia of aging (18). Ghrelin rises: plasma ghrelin was elevated in murine aging models (klotho-deficient, SAMP8), a finding not reproduced in humans, where reports show only decline or no change (19).

Two findings reconcile them, and both concern fidelity rather than level (**Table 1**). Rhythm: advanced age blunts postprandial ghrelin recovery, and frailty abolishes postprandial suppression entirely, thereby producing a loss of prandial rhythm in the frail elderly, based on a human clinical meal-test cohort (20). A hormone with a normal mean but no prandial excursion is decoupled from the fasted–fed transition — invisible to studies reporting means, decisive physiologically. Resistance: ghrelin treatment failed to stimulate appetite or prolong survival in klotho-deficient mice despite elevated endogenous ghrelin, indicating ghrelin resistance in aging (19). Elevated ghrelin with absent response is functionally equivalent to deficiency.

Consistent with both, older adults with a low-appetite phenotype showed greater ghrelin suppression after a standardized meal than young and appetite-matched older controls (21) - the opposite of simple deficiency. Cohort heterogeneity again contributes: undernourished older women had higher ghrelin than well-nourished older and young groups (18), indicating that nutritional status, not age alone, drives much of the between-study variance.

## 6 Controversy 3: lost hunger or excess satiety?

The excess-satiety school holds that heightened anorexigenic signaling suppresses intake. Intraduodenal lipid raises CCK more steeply in older men, plausibly slowing gastric emptying via increased pyloric motility (22); exogenous CCK-8 suppresses intake roughly twice as much in older as younger subjects while endogenous CCK is higher at baseline, argued as evidence that increased CCK activity causes the anorexia of aging (23); and this suppression is not attenuated by high-fat, high-energy supplementation (24). These are small, single-center comparisons of young versus older adults (n = 7–15 per group) using exogenous, supraphysiological CCK-8 infusions rather than physiological stimuli, so directionality of the age–CCK relationship cannot be established from them alone (22–24). The deficient-hunger school counters that in undernourished older women, reduced basal hunger rather than increased meal-induced satiety explained lower intake, with CCK and ghrelin changes deemed unlikely culprits (18). The composite school integrates across hormones: an anorexigenic response score combining ghrelin, GLP-1, and PYY responses was elevated in older adults with low appetite, suggesting that augmented anorexigenic responses to feeding are causal (21).

Meta-analysis of 35 studies (710 older, 713 younger adults) does not vindicate either position: older adults showed elevated fasting and postprandial CCK, leptin, and insulin, along with higher postprandial PYY, alongside lower energy intake and lower hunger in both fasted and postprandial states (25), though the constituent studies were themselves predominantly small and cross-sectional — as illustrated by (22–24) above — so the meta-analysis inherits, rather than resolves, this design limitation (25). Tonic and phasic anorexigenic signaling both rise. The most instructive detail comes from the low-appetite study: hormonal responses diverged markedly, whereas subjective appetite did not (21), implying the lesion lies in signal transduction rather than in perception - again, a coupling failure rather than a magnitude change (**Table 1**). Aging murine distal ileum also shows increased 5-HT synthesis and release, with reduced transporter-mediated reuptake, thereby elevating extracellular serotonin availability (26).

## 7 What the cellular literature adds, and where it conflicts?

The human disputes above are usually discussed without reference to a rapidly advancing rodent and *Drosophila* literature on age-related intestinal stem cell (ISC) mis-differentiation, and the two rarely cite each other. This is a problem, because they currently disagree (**Table 2**).

**Table 2:** Reported direction of enteroendocrine cell (EEC) number/output with age, by species and tissue.

Species/tissue	Finding	Direction with age	Candidate explanation for discrepancy
Human colon	Fewer L-cells; declining GLP-1 and PYY output (8, 9)	Decrease	Region- and species-specific niche signaling (31)
Human and mouse duodenum (nutrient-sensing/insulin receptor signaling)	Increased EECs, goblet cells, and Paneth cells; elevated chromogranin A (28)	Increase	Small-intestine vs. colon divergence; hormonal repertoire plasticity may inflate hormone-based counts (30)
Mouse ISC (IFNγ–Stat1 axis)	Lineage-skewed differentiation toward the secretory lineage; reversed by systemic IFNγ inhibition (27)	Increase	Inflammatory-niche driver not yet tested in human tissue
Aged mouse duodenum (Sox9-EGFP-High cells)	Increased reserve ISC/EEC population; reduced enteroid-forming capacity (29)	Increase	Consistent with (27, 28); human counterpart untested

That literature reports that aging mouse ISCs strongly upregulate antigen-presenting pathway genes and overexpress secretory lineage marker genes, resulting in lineage-skewed differentiation into the secretory lineage, driven by an IFNγ–Stat1 axis whose systemic inhibition completely reverses these aging phenotypes (27). Independently, dysregulated nutrient sensing distorts the direction of ISC differentiation via insulin receptor signaling, with enteroendocrine cells, goblet cells, and Paneth cells all increased, and chromogranin A elevated, in both the aged human and mouse duodenum (28). Aged mice likewise show increased numbers of Sox9-EGFPHigh cells (a population comprising reserve ISCs and enteroendocrine cells) alongside reduced enteroid-forming capacity (29).

The tension is plain: rodent small intestine gains EECs with age, while human colon loses L-cells and hormone output (8, 9) (**Table 2**). Region and species are obvious candidate explanations, and neither has been excluded. Two further considerations complicate any simple reading. EEC hormonal repertoire is plastic within lineage limits, such that a given cell can vary which hormones it expresses (30), so hormone-based counting may misestimate cell number. And EEC specification is governed by transcriptional networks that are demonstrably sensitive to environmental signaling (31), of which the aged niche provides ample. Whether the aged gut contains fewer EECs or a normal-to-increased number that are functionally miscalibrated remains unresolved and has not been tested with hormone-independent lineage tracing in aged tissue.

## 8 Discussion

Three controversies, one recurring resolution (**Figure 1**; **Table 1**). Incretin concentrations rise, and coupling fails; ghrelin levels may be preserved, and rhythm is abolished; anorexigenic hormones rise, and subjective appetite is unchanged. In each case, the dispute is sustained by measuring the amount, where the physiology depends on the relation. This is not merely a semantic reframing: it makes different predictions and demands different endpoints.

The therapeutic corollary is that interventions must be direction-specific, and GIP illustrates why this is not straightforward. Either an increase or a decrease in GIP secretion or action can lower body weight and glucose levels in preclinical models - a paradox resolved by tirzepatide’s biased agonism, which enhances GLP-1 receptor signaling by reducing internalization while functionally antagonizing the GIP receptor (32). For ghrelin, the reconciliation itself carries a therapeutic warning: because the deficit in aging is loss of rhythm and receptor responsiveness rather than reduced ghrelin availability (19, 20), simple ghrelin or ghrelin-mimetic replacement is unlikely to succeed, and restoring pulsatile secretion or GHSR1a sensitivity may be the more tractable target. For the anorexia of aging, the target is excess anorexigenic tone (25), with ghrelin potentiation a candidate (16, 19). For age-related dysglycemia, incretin mimicry dominates (32), but restoring endogenous secretion remains largely unattempted despite available tools: small molecules can drive human ISCs to hormone-secreting EECs (33), engineered ileal organoids permit live human L-cell study (34), and FXR signaling redirects ISC fate toward EEC differentiation (35).

Three gaps follow directly, in order of tractability. First, no study measures hormone concentration and functional coupling in the same aging individuals - the omission sustaining Controversy 1; this is readily addressable by adding an isoglycemic intravenous glucose arm to existing longitudinal cohorts such as that of Pham et al. (7). Second, the longitudinal design of Pham et al. (7) has not been extended to CCK, PYY, or ghrelin, leaving Controversies 2 and 3 dependent on cross-sectional data that are confounded by the entanglement of aging with diabetes, obesity, frailty, and nutritional status; a multi-hormone longitudinal panel in a single cohort would resolve much of this at modest incremental cost. Third, the human hormone literature and the rodent mis-differentiation literature must be reconciled (**Table 2**), beginning with region- and species-matched comparisons — the most resource-intensive priority, but the one most likely to determine whether the human anorexia-of-aging and T2D literatures should be interpreted through the same cellular lens as the rodent ISC-differentiation literature.

## 9 Conclusion

The aging gut is not simply a quieter endocrine organ. Its hormones are frequently present at normal or elevated concentrations while failing to carry the information they once did - losing prandial excursion, losing insulinotropic traction, losing correspondence with perceived appetite. Reading the literature this way converts an apparently contradictory evidence base into a coherent one and redirects effort toward endpoints that discriminate. The field should ask less often whether gut hormones rise or fall with age, and more often whether they still mean what they used to.
